# Microwave Radar-Based Cable Displacement Measurement for Tension, Vibration, and Damping Assessment

**DOI:** 10.3390/s26020494

**Published:** 2026-01-12

**Authors:** Guanxu Long, Gongfeng Xin, Zhiqiang Shang, Limin Sun, Lin Chen

**Affiliations:** 1 Shandong Key Laboratory of Highway Technology and Safety Assessment, Jinan 250098, China; 2 Innovation Research Institute of Shandong High-Speed Group Co., Ltd., Jinan 250098, China; 3 Department of Bridge Engineering, College of Civil Engineering, Tongji University, Shanghai 200092, China

**Keywords:** bridgecables, force identification, damping estimation, microwave radar, remote sensing, vibration characterization

## Abstract

Cables in cable-supported bridges are critical structural components with exceptional tensile capacity, and their assessment is essential for the safety of both the cables themselves and the entire bridge. Microwave radar, a non-contact and efficient measurement technique, has emerged as a promising tool for bridge cable evaluation. This study demonstrates the deployment of microwave radar on bridge decks to efficiently measure the displacements of multiple cables, enabling coverage of all cables while effectively eliminating low-frequency components caused by deck deformation and radar motion using the LOWESS method. The measured cable displacements can be directly used to characterize vibrations, particularly for detecting vortex-induced vibrations (VIVs), without the need for numerical integration of accelerations. Furthermore, microwave radar is applied to free-decay testing for cable damping evaluation, providing an improved signal-to-noise ratio and eliminating the need for sensors installed via elevated platforms, thereby enhancing the reliability of damping assessments. The effectiveness of these approaches is validated through field testing on two cable-stayed bridges.

## 1. Introduction

Cables are essential structural components with exceptional tensile load-bearing capacity, enabling the construction of long-span bridges, including cable-stayed bridges and suspension bridges. With the growing number of such bridges being built worldwide, cable-supported bridges are facing increasing challenges related to safety and durability throughout their service life. Among various structural parameters, cable tension under in-service conditions is one of the most critical indicators for assessing the safety and integrity of these bridges [1]. Moreover, due to their high flexibility in the transverse directions, cables are particularly prone to wind-induced vibrations [2], which can cause serviceability problems and lead to fatigue damage over the long term. Therefore, evaluating cable vibration characteristics and damping properties is of paramount importance for ensuring the long-term performance and maintenance of cable-supported bridges.

There are different mechanisms and corresponding methods for cable tension measurements, e.g., load cells or strain gauges installed at the cable anchorages [3]. These methods are direct approaches that can provide accurate cable force measurements but are often costly and labor-intensive, especially for large-scale cable-supported bridges with numerous cables. Alternatively, there are indirect methods based on cable profiles and vibration measurements [4]. Among them, vibration-based methods have been widely used in practice due to their advantages of being non-destructive, cost-effective, and reliable for long-term monitoring of cable forces. The fundamental principle of vibration-based cable force identification is based on the relationship between cable tension and its modal properties, which can be derived from the simple taut-string model or from more refined models that account for structural details of the cable, such as intermediate attachments and boundary conditions.

The development of the vibration-based cable force identification is closely associated with the advancements in sensing technologies for cable vibration measurements. At the early stage, traditional contact sensors, e.g., accelerometers, were used to measure cable vibrations at one point to extract the fundamental frequency to use the taut-string model for force evaluation, to extract multiple frequencies and then use the model of a tensioned beam for tension estimation. Today, this method is still popular for long stay cables whose dynamics can be accurately described by the taut-string model. With the advancements of sensing technologies, one can measure the cable dynamic responses to extract frequencies more efficiently and also more conveniently. The wireless accelerometer, laser Doppler vibrometer, microwave radar, and video-based methods have been used in practice, showing promise. The microwave radar is particularly attractive due to its capability of measuring multiple cables simultaneously from a distance without installing any targets on the cables [5,6]. A review of this technique will be provided in the subsequent section.

Later, to address the limitations of frequency-based methods for short cables or cables with intermediate supports, multiple accelerometers are proposed to measure the cable mode shapes [7,8]. Clearly seen in these theoretical and experimental studies is that if more information on cable statics and dynamics can be obtained, the accuracy of cable force identification can be further improved. Non-contact methods have also been employed to obtain more detailed information on cables, for example, by measuring cloud points to determine the cable static profile [9] or by capturing cable mode shapes of the cables through video-based methods [10], etc. These measurements require more advanced techniques and greater effort, and therefore have not yet gained widespread adoption in practice.

Given the promising potential of microwave radar for cable vibration measurement, its capabilities have not yet been fully explored. In this context, this study not only provides extensive field validation of microwave radar for efficient cable tension identification but also extends its application to bridge cable assessment, including vibration characterization and damping identification, which have not been examined in detail in the literature. The remainder of this paper is structured as follows. After the introduction, Section 2 reviews the state of the practice of microwave radar sensing for bridge cable assessments. Section 3 introduces the methods for cable displacement measurement, frequency-based cable force identification, cable damping estimation from free-decaying cable vibrations, and vortex-induced vibration detection of cables from microwave radar measurements. Section 4 presents the results of field tests on several cable-stayed bridges, including the CK Bridge and the ST Bridge. Finally, conclusions are drawn in Section 5.

## 2. A Brief Review of Microwave Radar Sensing for Bridge Cable Assessments

Microwave radar measures object displacement by detecting the phase difference of the transmitting and received microwaves, enabling simultaneous monitoring of multiple targets and significantly enhancing sensor deployment efficiency for large structures like bridge cables and girders. Early studies on this technology for bridges date back to [11,12]. Over the past two decades, numerous studies have investigated the use of microwave radar for bridge monitoring [13,14,15]. Notably, the review by [16] found that approximately 30% of microwave sensing studies focus on bridge applications, while extensive applications of microwave radar in other fields are also reported therein.

The need to measure the displacements of a large number of cables, both during regular inspections and for long-term monitoring in cable-supported bridges, aligns well with the capabilities of microwave radar systems. Consequently, applications and studies of microwave radar for bridge cable assessment have increased significantly in recent years. Early applications of microwave radar to bridge cables date back to the 2000s, with pioneering studies by Gentile and colleagues using the IBIS-S radar system [17,18,19,20,21]. These initial studies demonstrated the feasibility of radar-based cable monitoring on roadway bridges, such as the bridge crossing the River Adda between Olginate and Calolziocorte, cable-stayed bridges over the River Oglio and at Montodine in Italy, where tension forces of multiple cables were successfully measured with high accuracy compared to traditional contact sensors. An important focus of early studies was to validate microwave sensing against traditional accelerometers for cable vibration measurements.

Throughout the 2010s, the application of microwave radar expanded significantly across various bridge types. Gentile’s subsequent studies [5,22,23] demonstrated successful deployments on cable-stayed bridges and guyed masts for cable tension identification. Concurrent research by other groups [24,25,26] validated the technology on pedestrian bridges, on the cables of a hybrid cable-supported bridge [27] spanning the Ebro River at Amposta, Spain, and on suspender cables of suspension bridges or arch bridges [28,29], confirming the robustness of radar-based measurements across different cable configurations and bridge types. In addition to validation and comparisons based on field testing, Ref. [30] conducted experiments in both well-controlled laboratory settings and field applications on the Polcevera Viaduct in Genoa, Italy, using the IBIS-S radar system. Liu et al. [31] compared radar measurements with conventional sensors, including pressure sensors and strain gauges on CFRP cables, demonstrating comparable or superior performance.

In parallel with the application of microwave radar for cable displacement measurements, tailored algorithms have also been developed to process the large volumes of data generated. Ref. [32] proposed blind source separation and time–frequency analysis for separating cable signals located in the same range bin during the measurement, when applying microwave radar for 8 cables of the Nanjing Eye foot bridge. Ref. [33] applied time–frequency analysis for extracting time-varying tension force of the Hukun-Wuguang bridge induced by train passage. Weng et al. [4] developed a fully automated algorithm for frequency picking and mode order determination from displacement spectra for cable force identification using microwave radar systems, with applications to loading tests on a railway bridge.

Recent research has extended the applications of microwave radar to more complex cable geometries and loading scenarios, for example, on the Chongqing Nanjimen Railway Bridge [34], Shifeng Bridge, and Sanjiadian Bridge [35]. These studies have validated microwave radar performance on structures with large numbers of cables, demonstrating its scalability to massive cable systems. In most cases, radar measurements employed the real-aperture radar system, while two studies utilized the MIMO (Multiple-Input Multiple-Output) radar [6,36]. Notably, in the field measurements reported in [6], the MIMO radar enabled spatial resolution of cables on a bridge with closely arranged cables, allowing measurements of more than 30 cables from a single measurement station. Beyond using microwave radar for one-time cable force identification, there have been efforts to explore its use for structural assessment. For instance, Ref. [5] compared cable frequencies measured at different times using the IBIS-S radar for assessment. Ref. [37] compared IBIS-S measurements of a bridge in Porto Marghera, Italy, taken in 2011 and 2019. Similarly, Ref. [38] applied microwave radar on the Victoria Pedestrian Bridge under various loading conditions, including normal operation and dynamic events.

Apart from applications of microwave radar in cable force identification, many studies have focused on developing more portable, affordable, and capable devices (e.g., [6,29]). The use of microwave radar for long-term cable monitoring has only just begun. In short, its full potential for structural assessment of cables has yet to be explored.

## 3. Methods

In this section, several methods, respectively, for cable force identification, vibration characterization, and damping estimation are introduced. They will be used in the subsequent cases studies.

### 3.1. Microwave Radar for Cable Displacement Measurements

To measure all the cables of a cable-stayed bridge, it is preferable to deploy the microwave radar on the bridge deck. In this configuration, the measured raw signal also includes bridge deck deformations, particularly those induced by passing vehicles. To remove this influence, the Locally Weighted Scatterplot Smoothing (LOWESS) method is employed to separate the cable vibration displacements from the overall measured displacements. LOWESS is a non-parametric regression technique that combines multiple local regression models in a *k*-nearest-neighbor-based framework. It is especially effective for smoothing data and removing noise or unwanted trends.

Given a set of microwave radar displacement measurement points $\left(t_{j},u_{j}\right)$, $j=1,2,\ldots ,N_{t}$, the smoothed value at a point $t_{j}$ is obtained by fitting a weighted least-squares regression in a local neighborhood around $t_{j}$ expressed as

(1) $$\hat{u}\left(t_{j}\right)=\alpha_{j}t_{j}+\beta_{j},$$

where $\hat{u}\left(t_{j}\right)$ is the fitted curve, and $\alpha_{j},\beta_{j}$ are the coefficients of the local linear regression. The coefficients are estimated by minimizing

(2) $$\sum_{i=1}^{N_{t}}w_{i}{\left[u_{i}-\alpha_{j}t_{i}-\beta_{j}\right]}^{2}.$$

The weights $w_{i}$ decrease with the distance between time instances $t_{i}$ and $t_{j}$, typically using the tricube kernel function

(3) $$w_{i}\left(t_{j}\right)=\left\{\begin{array}{cc} {\left(1-{\left|\frac{t_{i}-t_{j}}{d(t_{j})}\right|}^{3}\right)}^{3}, & \mathrm{if}\;|t_{i}-t_{j}|<d\left(t_{j}\right)\\ 0, & \mathrm{otherwise}. \end{array}\right.$$

In the expression, $d(t_{j})$ is the distance from $t_{j}$ to its *q*-th nearest neighbor, with *q* determined by the smoothing parameter, which is the fraction of data used in each local regression.

After fitting the local model, Equation (1), the smoothed value is obtained. The cable displacement is thus obtained by subtracting the smoothed value from the original measurement as

(4) $$\tilde{u}\left(t_{j}\right)=u\left(t_{j}\right)-\hat{u}\left(t_{j}\right)=u\left(t_{j}\right)-{\hat{\alpha }}_{j}t_{j}-{\hat{\beta }}_{j},$$

where ${\hat{\alpha }}_{j},{\hat{\beta }}_{j}$ are estimated coefficients by solving the objective function as expressed in Equation (2).

### 3.2. Frequency-Based Cable Force Identification

In the case of using microwave radar to measure cable displacements, only one displacement is obtained for each cable. In this situation, frequency-based cable force identification methods are commonly used. The fundamental principle of frequency-based cable force identification relies on the relationship between the cable tension and its modal properties [4]. The following basic model relating cable tension and vibration frequencies is used,

(5) $$T=4mL^{2}{\left(\frac{f_{i}}{n_{i}}\right)}^{2}-EI{\left(\frac{n_{i}\pi }{L}\right)}^{2},$$

where *T* is the cable tension, *m* is the mass per unit length of the cable, *L* is the cable length, $f_{i}$ is the *i*-th identified natural frequency of the cable, $n_{i}$ is the mode number corresponding to the *i*-th natural frequency, EI is the bending stiffness of the cable cross-section. From one displacement time history measured by the microwave radar, only frequencies can be extracted, and therefore, the cable length and mass per unit length are normally assumed to be known and extracted from the structural drawings or design documents. Moreover, the bending stiffness term is often neglected for long cables where the bending stiffness effect is minimal, leading to a simplified formula with only the first term on the right-hand side, i.e., reduced to the taut-string model.

### 3.3. Vortex-Induced Vibration Detection of Cables

Stay cables are subjected to various types of wind-induced vibrations [2], including rain–wind-induced vibrations, dry cable galloping, and vortex-induced vibrations. The large-amplitude rain–wind vibrations occur in raining conditions and are relatively easy to distinguish, while the detection of high-frequency VIV is not straightforward. Recent studies have proposed various indices to detect VIV from cable vibration signals [39,40]. Most of the studies use accelerations as the original input signals. However, in this study, the cable displacement signals measured by microwave radar are shown to be effective for VIV detection as well.

Following the studies in [39,40], the measured cable dynamic response is used to construct the analytic signal z(t) through the Hilbert transformation,

(6) z(t)=u(t)+iv(t),

where i is the imaginary unit, and v(t) is the Hilbert transform of u(t). The Hilbert transformation H{ } is defined as

(7) $$v\left(t\right)=H\left\{u\right\}=h\left(t\right)*u\left(t\right)=\int_{-\infty }^{+\infty }u\left(\tau \right)h\left(t-\tau \right)d\tau =\frac{1}{\pi }\int_{-\infty }^{+\infty }\frac{u(\tau )}{t-\tau }d\tau ,$$

where h(t)=1/(πt) and τ is an integration variable.

Then, a VIV detection index can be defined based on the analytical signal. Here, the amplitude ratio following Ref. [39] is calculated based on the displacement amplitude envelope, as

(8) $$\mathrm{Rratio}=\frac{R_{\min}}{R_{\max}},$$

where $R_{\max}$ and $R_{\min}$ are the maximum and minimum of the envelope of the analytic signal, respectively. Note that a similar index is defined in [40] as $\left(R_{\max}-R_{\min}\right)/R_{\max}$ using cable accelerations. By analyzing the value of the index over time, VIV events can be effectively detected and characterized. A higher ratio indicates a more pronounced VIV event. The center coordinate is determined as [40],

(9) $$C_{R}=\mathrm{mean}\left(\sum_{i=1}^{N}R_{i}\right),C_{I}=\mathrm{mean}\left(\sum_{i=1}^{N}I_{i}\right),$$

where $R_{i}$ and $I_{i}$ are the real and imaginary parts of the analytic signal, respectively, and *N* is the total number of samples.

### 3.4. Cable Damping Estimation from Free Decaying Vibrations

For cable damping measurement, free-decay response analysis is arguably the most reliable approach. This requires the cable to be excited into a single-mode vibration and then left to decay freely. The resulting single-mode free-decaying vibration signal can be expressed as

(10) $$u\left(t\right)=Ae^{-\zeta_{n}\omega_{n}t}\sin\left(\omega_{d}t+\phi \right),$$

where *A* is the initial amplitude, $\omega_{n}$ is the natural frequency (rad/s), $\zeta_{n}$ is the damping ratio, $\omega_{d}=\omega_{n}\sqrt{1-\zeta_{n}^{2}}$ is the damped natural frequency, and ϕ is the phase angle. The amplitude envelope of the response is

(11) $$a\left(t\right)=Ae^{-\zeta_{n}\omega_{n}t}.$$

Taking the logarithm of Equation (11), the decay becomes linear in time,

(12) $$\ln a\left(t\right)=\ln A-\zeta_{n}\omega_{n}t.$$

Hence, the damping ratio can be estimated by performing linear regression on the logarithmic envelope of the cable displacement in the free-decaying period as

(13) $$\zeta_{n}=-\frac{1}{\omega_{n}}\cdot \frac{d\;[\ln\hat{a}\left(t\right)]}{dt},$$

where the slope of the fitted line in the semilogarithmic plot corresponds to $-\zeta_{n}\omega_{n}$. In this expression, $\hat{a}\left(t\right)$ denotes the envelope approximated from the measurement data, and d(·) is the differential operator.

In the previous studies, damping of bridge vibrations was computed from microwave measurements using stochastic subspace identification methods [30] from ambient excitation. Here, the free decaying response is used for damping estimation, which is typically used for cable vibration control design and validation [41,42].

## 4. Case Studies

### 4.1. Cable Force Estimation

#### 4.1.1. Description of the Bridge

**Figure 1:**
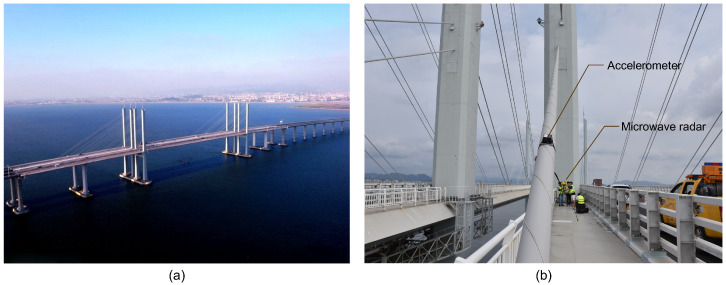
The CK Bridge for cable force identification: (**a**) photo of the bridge; (**b**) accelerometer and microwave radar deployment.

A cable-stayed bridge referred to as the CK Bridge was selected for field testing for cable force identification. The bridge is a twin-deck, separated twin-tower, dual-cable-plane steel box girder cable-stayed bridge, as shown in Figure 1. The bridge features auxiliary piers in the end spans and adopts a five-span continuous semi-floating structural system. The span arrangement is 80 + 90 + 260 + 90 + 80 m, with a spacing of 30.5 m between the separated tower legs and a tower column spacing of 20.6 m. The tower height is 82.5 m. The bridge is designed with a sparse-cable system, with two pairs of stay cables arranged in a harp-shaped configuration. A total of 96 stay cables are distributed across 16 cable planes. The stay cables are parallel steel-wire cables, and the steel wires used are 7 mm diameter galvanized high-strength, low-relaxation steel wires, with a tensile strength of 1670 MPa. The stay cables are sheathed in high-density polyethylene (HDPE) material, with the sheath surface treated with a helical pattern to reduce wind–rain-induced vibrations. The tensioning ends of the stay cables are anchored using cold-cast anchors.

#### 4.1.2. Field Testing Scheme

**Figure 2:**
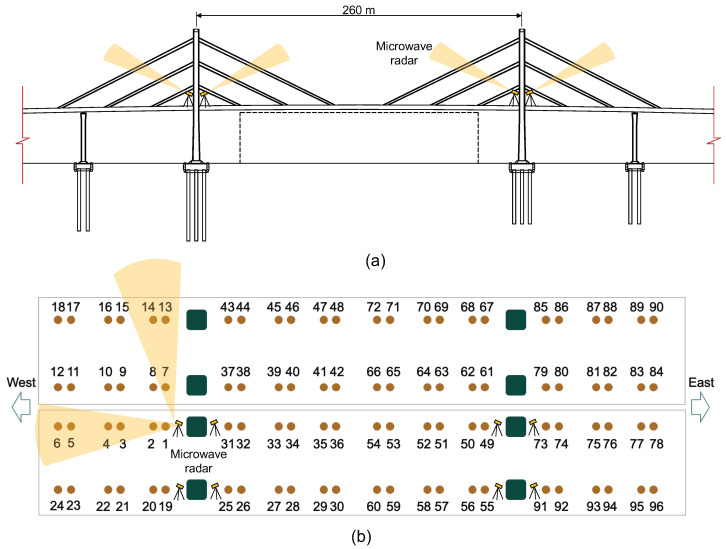
The field testing scheme for the CK Bridge: (**a**) the setup of the microwave radar; (**b**) the numbering of the cables.

To evaluate the performance and efficiency of the microwave radar for cable force identification, field tests were conducted on the bridge in September 2023. The microwave radar system used is the IBIS-FS developed by the IDS Company in Italy, as shown in Figure 1. During testing, the microwave radar was placed on the maintenance walkway or roadway near the bridge towers, with its lens directed toward the target cables, as shown in Figure 2. For comparison, wireless tri-axis accelerometers were installed on two selected cables to record cable vibrations simultaneously. The tri-axis accelerometer has a measurement range of ±10 g and a sampling frequency up to 2000 Hz. The microwave radar used in the test is also shown in Figure 1b, which has a measurement range of up to 1000 m, a displacement accuracy of 0.01 mm, a spatial resolution of 0.5 m, and a sampling frequency up to 200 Hz. Note that in the tests, only the bridge in the south side was accessible, and the cables of the north side bridge were measured from a longer distance when the radar was placed near the north tower of the south bridge. To cover all the cables, the orientation of the radar was adjusted at each measurement point.

During field testing, the installation and calibration of the radar at each measurement location took approximately 10 min, and each measurement was conducted twice, with each record lasting 5 min. For the whole bridge, the measurements of the 16 cable planes took a total of approximately 320 min, including 26 measurement cases. Figure 2b illustrates the numbering of the stay cables during the measurement process. Because the accuracy and consistency of the radar-based cable frequency measurement have been validated in the previous studies [5,18,31], accelerometers were only installed on Cable 1 and Cable 2 for comparison. The focus of the testing was to demonstrate the efficiency of the radar system for measuring all cables of a cable-stayed bridge.

#### 4.1.3. Results

**Figure 3:**
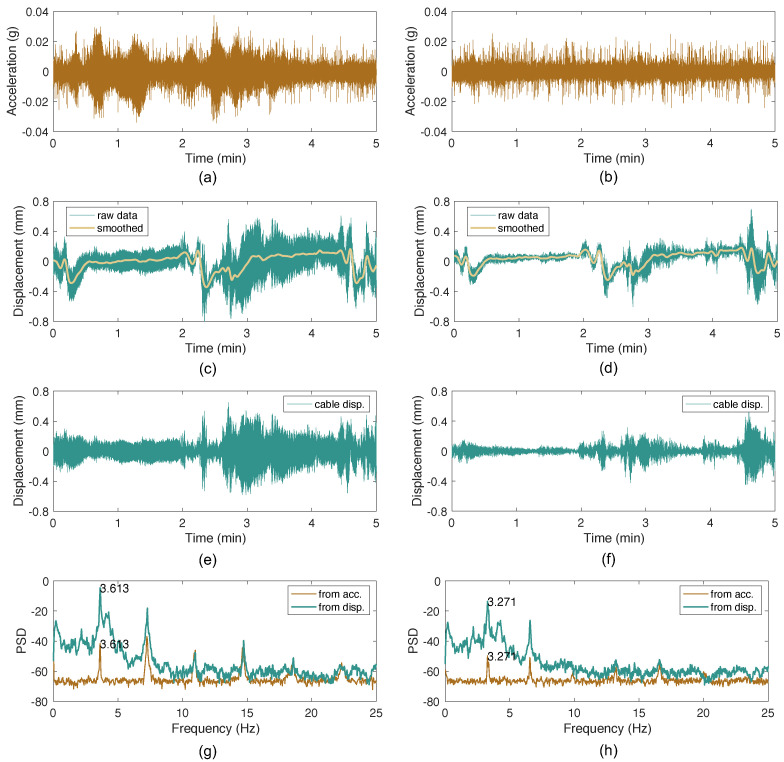
Comparison of frequency extraction from acceleration and displacements measured on two cables of the CK Bridge: (**a**) raw acceleration data of Cable 1; (**b**) raw acceleration data of Cable 2; (**c**) raw data from radar measurement on Cable 1 and the smoothed signal; (**d**) raw data from radar measurement on Cable 2 and the smoothed signal; (**e**) extracted displacement of Cable 1; (**f**) extracted displacement of Cable 2; (**g**) comparison of PSD curves for acceleration and displacement responses of Cable 1; (**h**) comparison of PSD curves for acceleration and displacement responses of Cable 2.

The measurement results of the two cables equipped with accelerometers are presented in Figure 3. The raw acceleration data of Cable 1 and Cable 2 are shown in Figure 3a and Figure 3b, respectively. The raw displacement data measured by the microwave radar for Cable 1 and Cable 2 are shown in Figure 3c and Figure 3d, respectively. It can be observed that the raw displacement data contain low-frequency components due to bridge deformations caused by passing vehicles or the motion of the radar head. After applying the LOWESS smoothing method, the smoothed signals effectively capture these low-frequency trends. By subtracting the smoothed signals from the raw displacement data, the dynamic displacement responses of the cables are obtained, as shown in Figure 3e,f. The power spectral density (PSD) curves of the acceleration and displacement responses for both cables are compared in Figure 3g,h. The fundamental frequencies extracted from both measurement methods are exactly the same, confirming the reliability of the microwave radar measurements.

**Figure 4:**
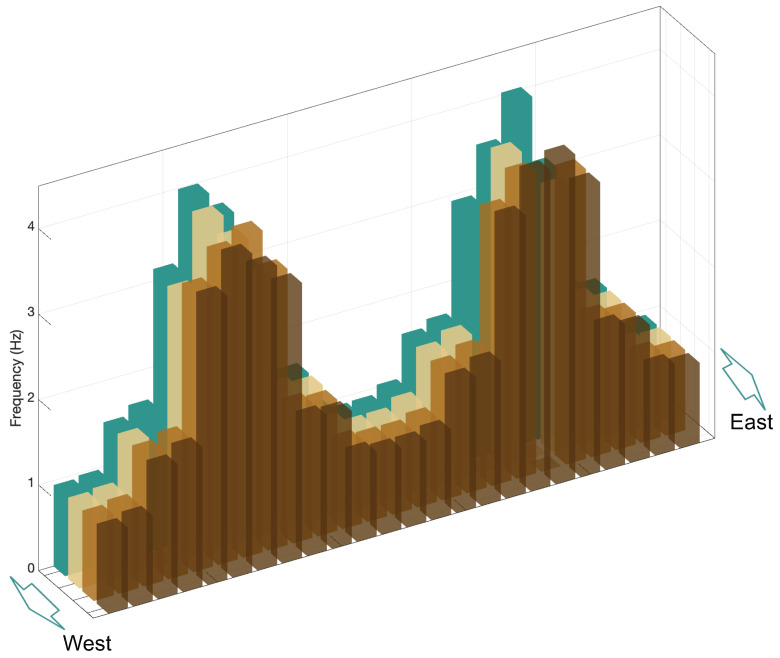
Extracted fundamental frequencies of the cables of the CK Bridge.

Using the processing procedure illustrated in Figure 1, the fundamental frequencies of the CK Bridge cables were extracted. The results are reported in Table 1 and plotted in Figure 4. Note that measurements taken at two different times using the microwave radar were consistent. Valid frequencies were obtained for 92 of the 96 cables; measurements for cables 9, 62, 73, and 79 were unavailable due to limited visibility and unfavorable observation geometry, and are therefore omitted. The frequency distribution in Figure 4 follows the bridge symmetry and the cable-length arrangement. Corresponding cables across the four cable planes (24 cables per plane) exhibit nearly identical fundamental frequencies, confirming the structural and geometric symmetry. In particular, the consistency of longer cables, the 64 cables further away from the towers, is notably high. Nevertheless, the shorter 32 cables closer to the towers show slightly larger variations in frequency, likely due to their shorter lengths and higher bending stiffness, making them more sensitive to local effects such as boundary conditions and possible damping devices located in the guide pipes of the cables.

After the fundamental frequencies were extracted, the cable tensions can be calculated using the frequency-based cable force identification method introduced in Section 3 together with cable properties obtained from structural design. As the taut-string model is used, the distributions and variations in the cable forces are similar to those of the frequencies. The cable force results are thus not presented here. Generally, this field testing demonstrates the efficiency and effectiveness of the microwave radar system for a large number of cables in cable-stayed bridges.

### 4.2. Vortex-Induced Vibration Detection from Microwave Radar Measurements

The field testing described in the previous cable tension identification was carried out with the cables under environmental excitations. The cable vibrations are generally of low amplitude. However, a further analysis of the measured cable displacement signals indicates that some cables experienced vortex-induced vibrations (VIVs) during the measurement period, as further detailed below. The VIV detection method introduced in Section 3 is used to analyze the measured cable displacement signals for VIV detection, following the recent studies [39,40].

**Figure 5:**
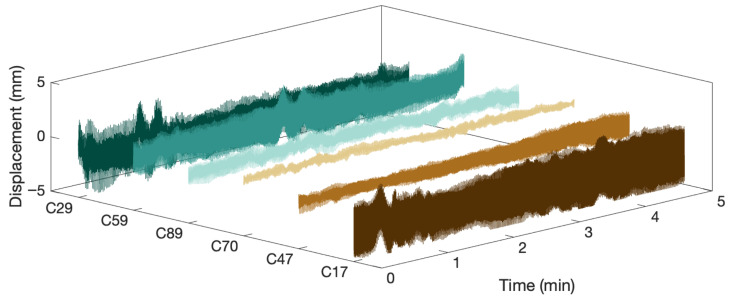
Typical displacement time histories measured using microwave radar on the CK bridge.

Figure 5 presents the measurement data from the six cables that exhibited relatively large vibration amplitudes during the test. The cable displacements are extracted by applying the LOWESS method to remove low-frequency components. Subsequently, the Hilbert transform is applied to each time history to obtain the corresponding analytic signal. For every one-minute segment, the analytic signal is then used to compute $C_{I}$, $C_{R}$, $R_{\min}$, and $R_{\max}$ following the procedure described in Section 3.

**Figure 6:**
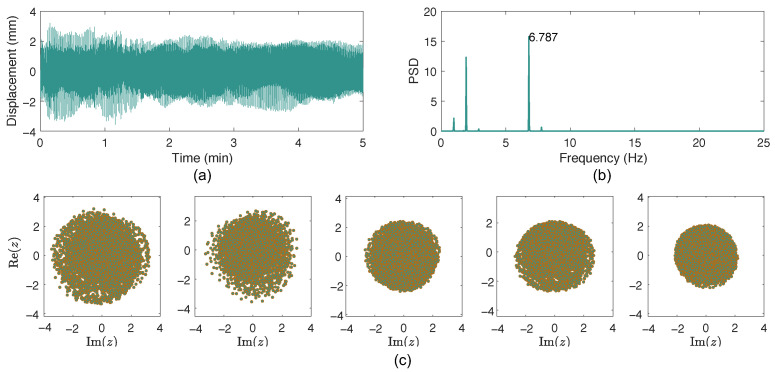
VIV detection of Cable 29: (**a**) cable displacement; (**b**) spectrum; (**c**) scatterplot of the corresponding analytical signal for each minute.

Figure 6 plots the analysis results of Cable 29. The spectrum suggests that the vibration has a dominant frequency of 6.787 Hz, corresponding to the 7th mode of the cable, while there are also other modes participating in the vibrations. The scatterplots of the analytical signals in Figure 6 clearly show that no distinct single-mode VIV occurred during the tests, as $R_{min}=0$ and Rratio = 0.

**Figure 7:**
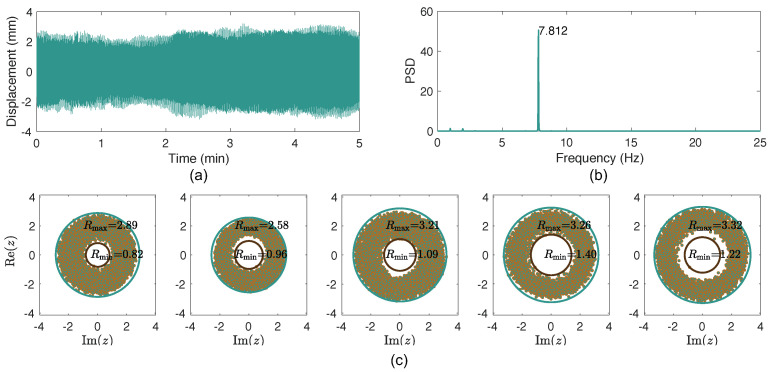
VIV detection of Cable 17: (**a**) cable displacement; (**b**) spectrum; (**c**) scatterplot of the corresponding analytical signal for each minute.

Figure 7 plots the analysis results of Cable 17. A dominant peak is identified in the spectrum plot in Figure 7b, corresponding to the 8th mode of the cable. In this case, the scatterplots of the analytical signals are quite different from those in Figure 6. For each minute, the values of $R_{\min}$ and $R_{\max}$ are also given in the figure. Correspondingly, the values of Rratio for the five minutes are 0.28, 0.38, 0.34, 0.43, and 0.37. Note that in Ref. [39], an Rratio equal to or large than 0.3 can be considered as the first-level warning condition of bridge girder VIVs.

It is noted that in [39], the bridge girder displacement was obtained through numerical integration of acceleration measurements for VIV detection. In contrast, Ref. [40] directly uses cable acceleration for cable VIV identification. With the microwave radar employed in this study, cable displacement can be directly measured at higher positions along the cable, enabling more reliable VIV detection than using acceleration measured near the lower end, and also allowing a larger number of cables to be monitored efficiently.

### 4.3. Cable Damping Assessment

#### 4.3.1. Description of the Bridge

**Figure 8:**
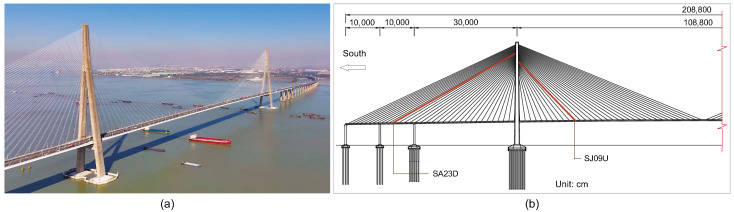
The ST Bridge: (**a**) the photo; (**b**) elevation plot and location of the tested cables.

Figure 8 shows the landmark cable-stayed bridge in China, referred to as the ST Bridge. The bridge has a total length of about 8206 m and a main span of 1088 m. Featuring inverted Y-shaped concrete pylons reaching 306 m high and a streamlined steel box girder deck, the bridge girder has a width of 33.5 m and carries six lanes of traffic. A total of 272 stay cables support the bridge deck, arranged in a fan-shaped pattern from the pylons to the deck edges, with cable lengths ranging from 150 m to 577 m.

#### 4.3.2. Field Testing

**Figure 9:**
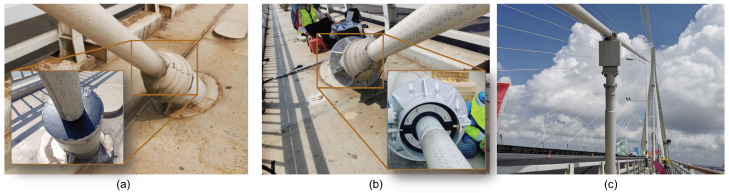
Photos of the dampers installed on the two cables: (**a**) internal damper on SJ09D; (**b**) internal damper on SA23D; (**c**) external damper on SA23D.

Because of the length and flexibility of the cables of the bridge, cable vibration control has been a key consideration in the bridge design and maintenance [41,42]. The field testing was aimed at performance evaluation of the cables attached with different types of dampers for vibration control. Specifically, two cables, denoted as SJ09D and SA23D, were selected for the damping identification tests. The location of the two cables is shown in Figure 8b, and they are on the downstream side of the bridge, i.e., east side. Cable SJ09D is equipped with an internal high-damping rubber damper while cable SA23D is installed with both an external damper and an internal high-damping rubber damper, respectively, for wind–rain vibration control and high-order VIV control, as shown in Figure 9. The parameters of the two cables are listed in Table 2.

**Figure 10:**
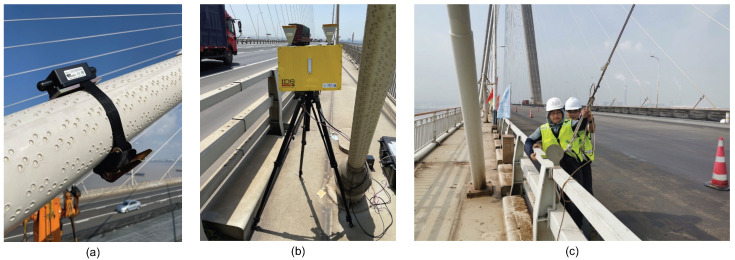
Photos of the field testing: (**a**) tri-axis accelerometer; (**b**) microwave radar; (**c**) manual excitation.

The accelerometers and microwave radar used were the same as for the cable force identification in Section 4.1. The installation of the tri-axis accelerometer on the cable is shown in Figure 10a, and the microwave radar setup is shown in Figure 10b. The radar was placed on the bridge deck. Figure 10c shows the manual excitation during the tests, where the cable is pulled laterally by hand to initiate free decaying vibrations in a single mode.

For each of the two cables tested, the accelerometer was first installed at a height of approximately 10 m above the bridge deck using an elevated work platform. A wire rope was then looped around the stay cable and pulled upward to a higher position to perform manual excitation. The microwave radar was deployed on the bridge deck and oriented toward the target cable at the corresponding higher location. The data acquisition system was then turned on. First, the cable responses under ambient excitation were measured and analyzed to identify the cable frequencies, which were then used to excite the cable mode by mode. During manual excitation, the wire rope was pulled and released according to the target cable frequency. Manual excitation is difficult below 0.5 Hz or above 3 Hz due to limitations in human force and timing. After successfully exciting the target cable mode, the excitation was stopped, allowing the cable to freely decay.

#### 4.3.3. Testing Results and Discussions

**Figure 11:**
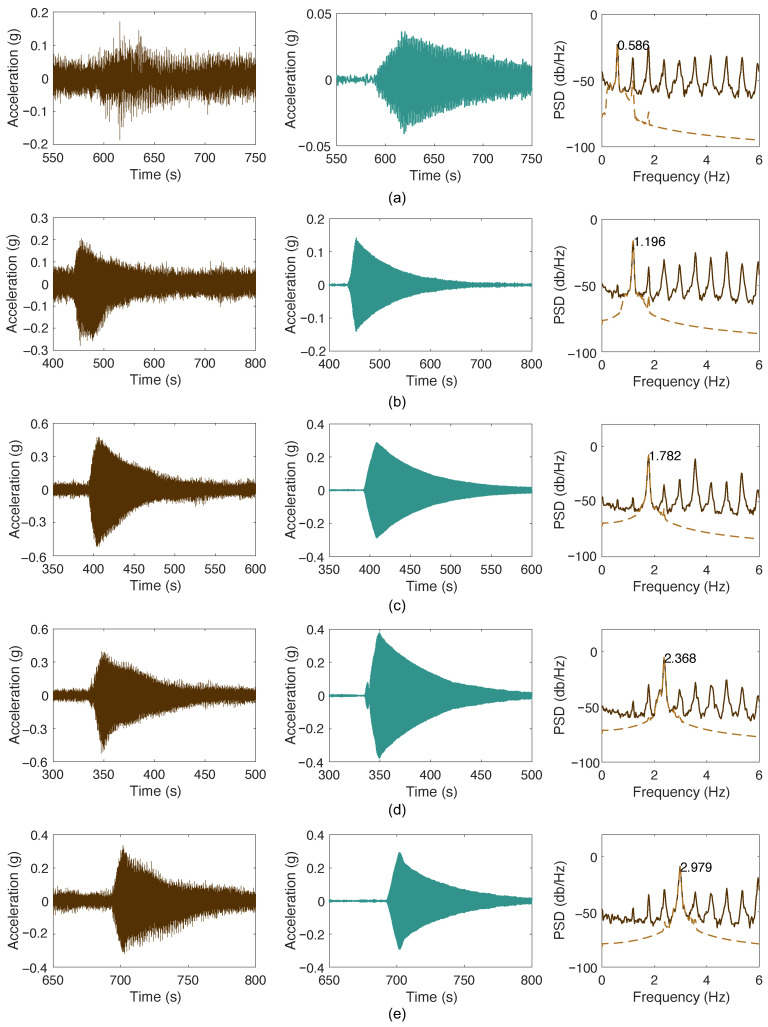
Measured accelerations of cable SJ09D and its spectra: (**a**) mode 1; (**b**) mode 2; (**c**) mode 3; (**d**) mode 4; (**e**) mode 5.

The measured results on cable SJ09D are first presented. Figure 11a illustrates the raw accelerations measured, respectively, in the tests targeting the first five modes. The measured signals contain both the target mode and contributions from other modes, as shown in Figure 11b. The target mode is not clearly dominant because the excitation and sensor placement are near the cable anchorage. Therefore, the accelerations were filtered using a bandpass filter centered at the cable frequency with a 0.8 Hz bandwidth, resulting in a signal that predominantly contains a single-mode vibration, as shown in Figure 11c.

**Figure 12:**
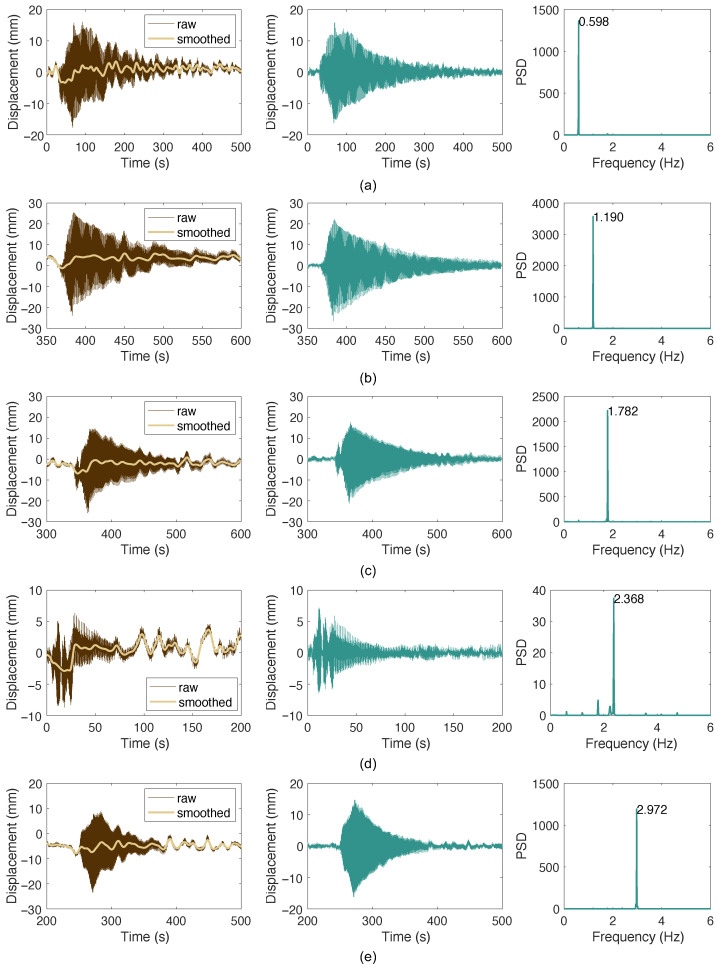
Measured displacements of cable SJ09D and its spectra: (**a**) mode 1; (**b**) mode 2; (**c**) mode 3; (**d**) mode 4; (**e**) mode 5.

Figure 12 presents the raw measurement data obtained from the microwave radar. The data show a clear excitation and decaying response in the cable vibrations, even for the first mode. Also shown is the signal with low-frequency components removed using LOWESS. The corresponding spectrum clearly highlights the dominant peak of the target cable mode. This is because the microwave radar measures the cable response at a higher position, where the vibration amplitude is larger, resulting in an improved signal-to-noise ratio.

**Figure 13:**
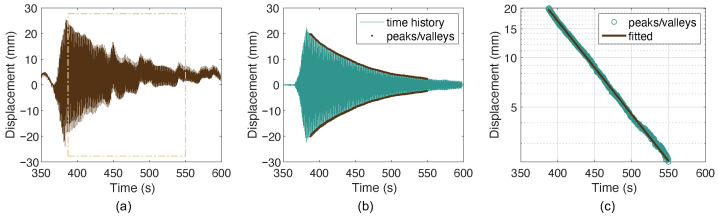
Example of damping computation from the measured cable response: (**a**) selection of free-decaying response; (**b**) filtering and peak/valley picking; (**c**) envelope fitting.

The frequencies and damping ratios of cable SJ09D were then extracted from the filtered accelerations and the displacements. The procedure involves applying a time window to isolate the cable’s free-decaying response, identifying the peaks and valleys of the signal, and performing curve fitting, as illustrated in Figure 13.

Table 3 lists the frequencies, damping ratios, and corresponding logarithmic decrement values for all five modes identified from the field tests. The results obtained from the accelerometer and the microwave radar are in good agreement, with the frequencies of each mode nearly identical. The damping ratios also show reasonable consistency, although some variation is expected due to the inherent challenges in damping estimation. Overall, the logarithmic decrement values for the first five vibration modes are approximately 0.01, corresponding to a Scruton number of around 5, which is sufficient to control daily vortex-induced vibrations.

**Figure 14:**
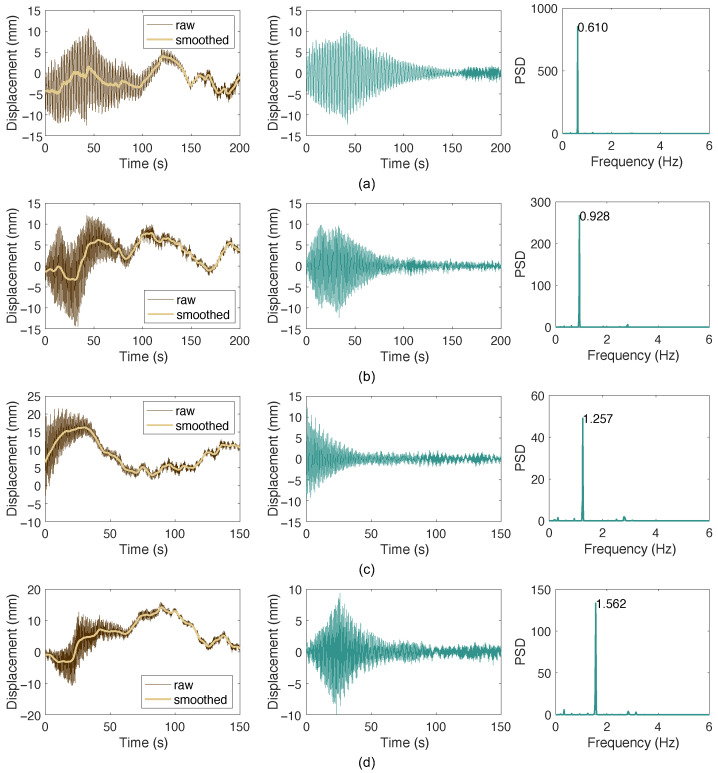
Measured cable responses: (**a**) mode 2; (**b**) mode 3; (**c**) mode 4; (**d**) mode 5.

For cable SA23D, vibrations from the 2nd to the 9th modes were successfully excited on-site. The first-mode vibration could not be excited due to the cable’s large length and low fundamental frequency of approximately 0.3 Hz. The acceleration data are similar to those of SA09D and are therefore omitted for conciseness. The microwave radar was used to measure the 2nd to 5th modes, with the results shown in Figure 14, exhibiting characteristics similar to those in Figure 12. The data processing procedures and methods are identical to the previous case. Table 4 summarizes the frequencies, damping ratios, and corresponding logarithmic decrement values calculated from the acceleration and displacement responses. The results for the 2nd to 5th modes are generally consistent between the two methods. Furthermore, the table indicates that the damping measurements meet design requirements, with logarithmic decrement values for the first five modes exceeding 0.03 and other modes achieving a Scruton number greater than 10 (equivalent to a modal damping ratio of approximately 0.32%) [41,42].

## 5. Conclusions

In this study, microwave radar is employed for bridge cable assessment, including cable tension identification, vibration characterization, and damping estimation. Through field testing and data processing, the following conclusions can be drawn.

- The microwave radar can be deployed on the bridge deck to measure the displacements of multiple cables in cable-stayed bridges, greatly improving measurement efficiency and enabling coverage of all cables. Low-frequency components caused by bridge deck deformation and radar head motion can be effectively removed using the LOWESS method.
- Cable displacements measured by the microwave radar can be directly used to characterize cable vibrations, particularly for detecting vortex-induced vibrations. This approach avoids the need for numerical integration of acceleration data to obtain displacements and allows monitoring of more cables.
- Using the microwave radar for free-decay testing to evaluate cable damping offers several advantages. First, there is no need to install sensors via an elevated platform. Secondly, the signal-to-noise ratio of the measured data is improved, and thus, the reliability of damping evaluation is enhanced.

Although this study demonstrates that microwave radar is advantageous in the presented applications, measuring the mode shapes of the cables using microwave radar is difficult, if not impossible. Therefore, microwave radar is not suitable for accurate cable force identification of short cables when mode shapes are required.

## Figures and Tables

**Table 1:** Field testing results of the cables of the CK bridge.

Cable No.	Frequency (Hz)	Cable No.	Frequency (Hz)	Cable No.	Frequency (Hz)	Cable No.	Frequency (Hz)
18	0.951	12	0.950	6	0.945	24	0.936
17	0.979	11	0.976	5	0.987	23	0.994
16	1.516	10	1.531	4	1.539	22	1.513
15	1.634	9	-	3	1.613	21	1.629
14	3.142	8	3.090	2	3.271	20	3.315
13	3.993	7	3.880	1	3.613	19	3.725
43	3.687	37	3.527	31	3.764	25	3.502
44	2.990	38	2.996	32	3.231	26	3.215
45	1.599	39	1.625	33	1.583	27	1.583
46	1.557	40	1.556	34	1.533	28	1.547
47	1.047	41	1.039	35	0.983	29	0.989
48	0.923	42	0.929	36	0.937	30	0.945
72	0.929	66	0.929	54	0.934	60	0.943
71	1.032	65	1.030	53	0.994	59	0.993
70	1.542	64	1.535	52	1.519	58	1.519
69	1.632	63	1.644	51	1.623	57	1.625
68	2.929	62	-	50	3.181	56	3.248
67	3.505	61	3.616	49	3.525	55	3.693
85	4.029	79	-	73	-	91	3.792
86	3.120	80	3.035	74	3.393	92	3.383
87	1.648	81	1.666	75	1.611	93	1.639
88	1.541	82	1.542	76	1.546	94	1.546
89	0.973	83	0.973	77	1.009	95	1.006
90	0.943	84	0.944	78	0.938	96	0.934

**Table 2:** Cable properties and theoretical analysis of the vibration frequencies.

No.	Length (m)	Tension (kN)	Mass (kg/m)	1st Mode Freq. (Hz)	2nd Mode Freq. (Hz)	Ext. Damper Position (%)	Int. Damper Position (%)
SJ09D	212.51	3005.5	48.5	0.588	1.176	-	1.06
SA23D	412.82	4684.0	72.6	0.308	0.616	2.33	0.89

**Table 3:** Field testing results of cable SJ09D.

Mode No.	Accelerometer Measurement			Microwave Radar Measurement		
	Freq. (Hz)	Damping (%)	Log. Decr.	Freq. (Hz)	Damping (%)	Log. Decr.
1	0.586	0.252	0.0158	0.598	0.239	0.0150
2	1.196	0.179	0.0113	1.190	0.176	0.0110
3	1.782	0.148	0.0093	1.782	0.147	0.0092
4	2.368	0.176	0.0111	2.368	0.203	0.0128
5	2.979	0.150	0.0094	2.972	0.126	0.0079

**Table 4:** Field testing results of cable SA23D.

Mode No.	Accelerometer Measurement			Microwave Radar Measurement		
	Freq. (Hz)	Damping (%)	Log. Decr.	Freq. (Hz)	Damping (%)	Log. Decr.
2	0.613	0.532	0.033	0.610	0.614	0.038
3	0.928	0.557	0.035	0.928	0.631	0.040
4	1.221	0.535	0.034	1.257	0.592	0.037
5	1.538	0.490	0.031	1.562	0.562	0.035
6	1.856	0.393	0.025	-	-	-
7	2.173	0.412	0.026	-	-	-
8	2.490	0.376	0.024	-	-	-

## Data Availability

The data presented in this study are available on request from the corresponding authors.
